# Notes on the data quality of bibliographic records from the MEDLINE database

**DOI:** 10.1093/database/baad070

**Published:** 2023-11-04

**Authors:** Robin Bramley, Stephen Howe, Haralambos Marmanis

**Affiliations:** Copyright Clearance Center Limited, Ivory House, St Katharine Docks, London E1W 1AT, UK; Copyright Clearance Center Inc., 222 Rosewood Drive, Danvers, MA 01923, USA; Copyright Clearance Center Inc., 222 Rosewood Drive, Danvers, MA 01923, USA

## Abstract

The US National Library of Medicine has created and maintained the PubMed® database, a collection of over 33.8 million records that contain citations and abstracts from the biomedical and life sciences literature. This database is an important resource for researchers and information service providers alike. As part of our work related to the creation of an author graph for coronaviruses, we encountered several data quality issues with records from a curated subset of the PubMed database called MEDLINE. We provide a data quality assessment for records selected from the MEDLINE database and report on several issues ranging from parsing issues (e.g. character encodings and schema definition weaknesses) to low scores for identifiers against several data quality metrics (e.g. completeness, validity and uniqueness).

**Database URL**  https://pubmed.ncbi.nlm.nih.gov

## Introduction

PubMed is an enormously valuable resource for the biomedical and health fields. The PubMed database is a voluminous collection of medical literature citations, which is free and easily accessible and has been a data source for many works in the information retrieval and life sciences communities. As machine learning becomes more prevalent in various branches of life sciences, the number of works that rely on the PubMed database increases. Many papers that cited PubMed have appeared within the proceedings of The International Conference on Data and Text Mining in Biomedicine series, e.g. DTMBIO '10 (1). In ACM’s Digital Library (2), the year 2021 was a new high point at 235 for computing research articles that mentioned PubMed in the full-text collection, up from 1 in 1998 to 115 in 2010. Many information providers utilize the PubMed database, and there are a variety of machine learning models trained on PubMed (3). It should be no surprise that, during the Coronavirus disease 2019 (COVID-19) pandemic, the PubMed database has been crucial in providing timely and frictionless access to the scientific literature (4).

However, the PubMed database, which contains over 33.8 million records (5) collected over many decades, suffers from several data quality issues. These issues relate to, in part, character encodings, the absence of persistent identifiers, differences in human languages and schema changes. These shortcomings should not be surprising since PubMed aggregates information produced by different publishers and extensible markup language (XML) data providers, a fact that leads naturally to the presence of ‘multi-source problems’ (6).

MEDLINE is a curated subset of PubMed, and its records are indexed with a controlled vocabulary called Medical Subject Headings (MeSH) (7) and include information regarding funding, genetic, chemical and other metadata. Articles in MEDLINE predominantly come from a set of indexed journals, and a reference data file of these journals is available separately (8). MEDLINE was made available online, through PubMed, in 1997.

While PubMed is a bibliographic database, National Library of Medicine (NLM) also provides access to archived full-text articles through PubMed Central, which was launched in 2000. There are 9 million articles archived, including the BioC subset (9) (nearly 3 million articles in 2019) that utilizes a simplified XML structure specifically designed for text mining.

In this article, we will provide an account of our experience in working with the curated MEDLINE records and report on the data quality issues that we encountered. We will describe, at length, the problem of author name disambiguation, which is widely acknowledged as a source of error when processing bibliographic databases in general, due to the challenges of synonyms (e.g. ‘John Doe’, ‘John T Doe’ and ‘JT Doe’ referring to the same individual) and homonyms (i.e. two different people who share the same name such as ‘John Smith’). Sanyal *et al.* (10) provide a review of author name disambiguation techniques for PubMed, which includes the influential ‘Author-ity’ model (11) and the work that the US NLM has undertaken to disambiguate authors through the downstream PubMed search engine (12) to reduce the impact on front-end users.

Other problem areas that we will discuss include issues with character encodings, date-related issues, the presence of persistent identifiers (and lack thereof), affiliation disambiguation, language-related data issues and schema data quality issues. Knowing how to address these challenges is valuable for practitioners who need to work with MEDLINE (or databases like MEDLINE) and process its records so that they can be used in their information systems.

### PubMed data

The PubMed database is available as XML, with the grammar described using a document type definition (DTD), the 2019 version at the time of execution (13). The compressed files are made available via an (file transfer protocol (FTP) server (they are also accessible by hypertext transfer protocol secure, and each one of them contains up to 30 000 citation records. Every year, in mid-December, the data are consolidated and an annual baseline is produced. This is followed by incremental daily update files that include deletions.

A PubMed XML file has a root element of PubmedArticleSet that contains one or more PubmedArticle or PubmedBookArticle children. The DTD also permits 0 or 1 DeleteCitation elements, and these can be seen in the update files. The elements of the PubmedArticle are divided into the MedlineCitation and the optional PubmedData—we have colloquially referred to these as the ‘front’ and ‘back’ matter, respectively. Each record is identified by a unique PubMed Identifier (PMID).

The description of the XML elements (14) also outlines the potential discrepancies caused by schema changes or policy changes to the collected data. For example, records created before 2002 only contained author initials instead of full, first or middle names; moreover, records between 1988 and 2013 only included the affiliation for the first author.

#### Known DTD shortcomings

There are two known problems with the DTD that have not yet been addressed. The first known problem is that authors cannot be linked to their CollectiveName (e.g. a working group). Some publishers have tried to work around this by interspersing CollectiveName elements and Author elements. In a wheat genome sequencing consortium paper (PMID 30115783), one of the contributors was a member of 12 groups, so that person appeared as an Author record 12 times. This multiplicity complicates the author name disambiguation, as it may be impossible to distinguish a duplicate author entry from a valid homonym.

The second problem is related to a shortcoming in the 2019 DTD. Specifically, the back matter PubmedData element may contain a ReferenceList with many Reference elements, but it does not prevent the presence of many ReferenceList elements each with one Reference. Consequently, extraction must be able to handle both because both have been observed in the records. Furthermore, the ReferenceList definition permits deeply nested ReferenceList elements, as shown below:

<!ELEMENT ReferenceList (Title?, Reference*, ReferenceList*)>

#### Escape characters

Escape sequence characters may appear within text fields such as the article title or abstract text. For example, if you wanted to represent a record in JavaScript Object Notation (JSON), then you would have to be aware of trailing backslashes and double quotes. Backslashes can also be problematic for the language used to parse the record. Furthermore, it may be necessary to remove other special characters such as new line characters (e.g. carriage return and line feed), tabs and so on.

#### Extended characters

PubMed encompasses articles published in many different languages, sometimes multiple languages. Consequently, fields such as the affiliation string, or parts of the author’s name, may contain extended characters. This is an important consideration for the disambiguation of author names or for matching organizations or places in affiliation strings (e.g. Istanbul vs. İstanbul).

### Open-source libraries

Since PubMed has been a canonical source of biomedical citations, there are open-source libraries to assist with parsing the records. While none of these libraries were appropriate for our needs, they are included here for completeness.

For Python, Pubmed Parser (15) is an active project, but it only handles a constrained field list. The pymed (16) project, which is now archived, only parsed and cleansed a limited subset of the fields. It also seems that the design was intended to wrap the PubMed application programming interface.

For Java, there is Pubmed Parser (17), which is based on the Java Architecture for XML Binding. This project only had a short flurry of commits over 6 days in April 2021, and consequently, it is unclear whether this is actively maintained.

## Materials and methods

This work will identify challenges that can be faced when working with the MEDLINE data and categorize them along several dimensions of data quality (18).

### Data acquisition

The PubMed baseline files were downloaded from their respective NLM FTP folders (19, 20) and uploaded to separate folders on an S3 bucket.

### Data processing

**Figure 1.**
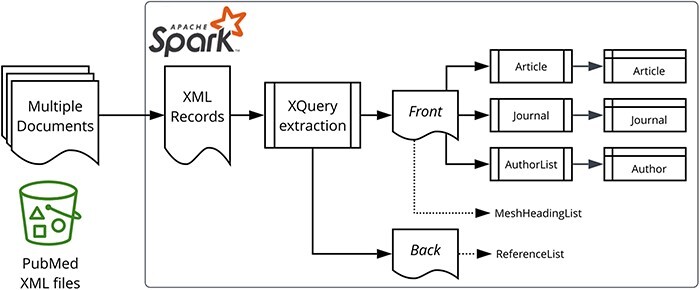
Data processing overview. Apache Spark and the star logo are trademarks of the Apache Software Foundation in the United States and/or other countries.

Figure 1 illustrates our data processing approach. The PubMed-gzipped XML files were processed using Apache Spark™ 3.1.1 (21) on Amazon EMR 6.3.1. The initial ingestion process extracted a few key properties, such as the PMID and digital object identifiers (DOI) (from the PubmedData if present), before splitting the XML into two fragments representing the front matter (bibliographic metadata) and the back matter (references).

The baseline files were ingested first, and then the update files were subsequently processed to apply updates, insertions and deletions. Record updates were applied by sorting the records by their PMID in conjunction with the DateRevised property; only the newest records were retained. Note that the PMID Version attribute is not suitable for this purpose as it is only used by Public Library of Science records (14).

Spark SQL (22) is designed for tabular data, with the key construct being the DataFrame, while XML documents are represented using a hierarchical structure that allows for repeating elements (a one-to-many relationship). This leads to an inherent mismatch between the two data formats that requires data transformation.

There is a spark-xml module (23), but we discovered during our initial experiments that the PubMed XML was too complex for spark-xml, as it resulted in heavily nested DataFrames and incorrect query results. Consequently, we solved the XML to DataFrame impedance mismatch by performing an XQuery (24) operation per target entity type (e.g. Article, Author, etc.) as shown on the right-hand side of Figure 1.

The spark-xml XmlInputFormat class was retained for loading the XML files into Spark, with the ingestion and extraction utilizing XQuery queries to extract properties, via the Saxon-HE (25) library as provided by the Elsevier Labs spark-xml-utils (26) module.

**Figure 2.**
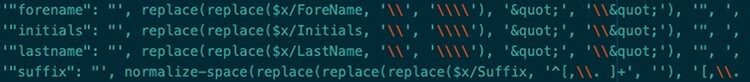
JSON representation within XQuery.

To ease the maintenance of the complex XQuery queries, we adopted a pattern whereby the XQuery output produces a JSON document. This makes the target property for a particular XPath or XQuery expression transparent (Figure 2), and inserting new elements does not break downstream code because it does not rely on positional information. The last part of that transformation phase is to leverage the read method of the SparkSession object, which parses the JSON documents to DataFrame records. Note that Figure 2 also represents the handling of escape characters (as described in the Escape characters section) using the XQuery replace function.

### Data analysis

The resulting DataFrames were analyzed using Spark SQL in Apache Zeppelin (27). For string fields, we consider the length in characters and in words (by splitting on spaces). Metrics were rounded to three decimal places (or less).

The plots were produced using ggplot2 (28) in R (29), with the box plots using the log scale for the *y*-axis.

### Identifier metric definitions

For the identifiers, such as the DOI (30) for an article, the data quality dimensions (18) that will be considered are their ‘completeness, validity and uniqueness’. These can be defined as follows:

-
$\mathcal{N}$ = number of records
-
$\mathcal{M}$ = number of records missing a value for the target property
-
$\mathcal{D}$ = distinct values of those present (excludes null/blank)
-
$\mathcal{V}$ defined by the count of records matching a regex for identifiers (Table 1)
-
$\mathcal{P}$ = present = $\mathcal{N}$—$\mathcal{M}$
-
$Completeness$ metric = $\mathcal{P}$/$\mathcal{N}$
-
$Validity$ metric = $\mathcal{V}$/$\mathcal{P}$
-
$Uniqueness$ metric = $\mathcal{D}$/$\mathcal{P}$

**Table 1.** Regular expressions for identifier validation

Identifier	Regular expression
DOI	‘^10.\d{4,9}/[-._;()/:a-zA-Z0-9]+$’ ^a^
ORCID (31)	‘^\d{4}-\d{4}-\d{4}-(\d{3}X|\d{4})’
ISNI (32) (presentation)	‘[0-9]{4} [0-9]{4} [0-9]{4} [0-9]{3}[0-9X]’
ISNI (compact)	‘[0-9]{15}[0-9X]’
GRID (33)	‘grid\.\d{4,6}\.[0-9a-f]{1,2}’


a Adapted from https://www.crossref.org/blog/dois-and-matching-regular-expressions/.

### Limitations of the study

The source dataset comprises the PubMed 2022 baseline plus daily update files up to 1252 (30 March 2022); NLM advises that these data do not reflect the most current/accurate data available from NLM.

It should be noted that our study includes only the PubmedArticle records, not the PubmedBookArticle records. The PubmedArticle records are only those from the MEDLINE subset (based on the Status attribute) and further exclude news articles and those articles without a title; this gives a total of 28 986 590 article records. News articles were excluded from extraction because journalists, such as Gareth Iacobucci from the British Medical Journal with over 1500 records, skew attempts to identify prolific authors through aggregation.

Other applied constraints are as follows:

- Only Author records with the ValidYN attribute of Y have been extracted, not Investigator records. For these 120 191 520 authors, only the first Affiliation element is considered.
- Open Researcher and Contributor ID (ORCID) and International Standard Name Identifier (ISNI) were checked using regular expressions rather than verifying the checksum digit. Therefore, well-formatted but erroneous ORCIDs would be incorrectly treated as valid, although 0000–0000-0000-0000 which occurs 5 times was explicitly detected. The other case that is not handled is where a publisher has reported the same ORCID for different authors, e.g. 0000–0002-5696-5368 was assigned for both Omer Eker and Carlos Riquelme on PMID 29382772.
- The DataBank element provides links to external datasets such as clinical trials. These identifiers were not investigated as part of the reported study.
- For alternative article identifiers, we did not extract the ELocationID element nor Publisher Item Identifiers from the PubmedData.
- For journals, International Standard Serial Number were not analyzed.

Lastly, it should be noted that for string fields outlier detection, e.g. by length, will highlight certain data issues, but it will not capture all cases.

#### Approximation

Five-number summary information is produced using the Spark’s DataFrameStatFunctions approxQuantile method with an error margin of 0.0001, an example is shown below:

articleDF.stat.approxQuantile(“doi_len”, Array(0.0,0.25,0.5,0.75,1.0), 0.0001).

However, the distinct counts do not leverage the Spark SQL approx_count_distinct function, rather the dataframe.select(“column”).distinct.count approach was used.

## Results and discussion

**Figure 3.**
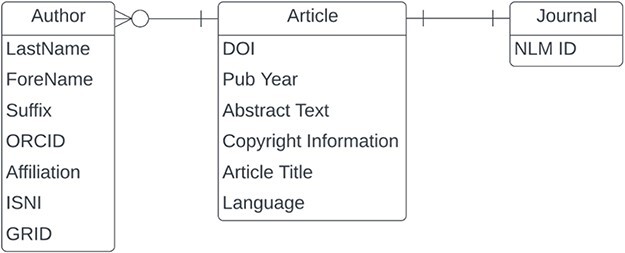
Entity relationship diagram for a subset of PubMed.

In this section, we will present our results related to data quality for the entities and fields shown in Figure 3. The PubMed XML data model is article-centric, but we will work our way from left to right.

### Data quality issues related to author names

One of the important considerations regarding author records is that PubMed has not always recorded all the authors of a paper. The number of authors was limited to 10 between the years 1984 and 1995 and to 25 between the years 1996 and 1999 (14).

**Figure 4.**
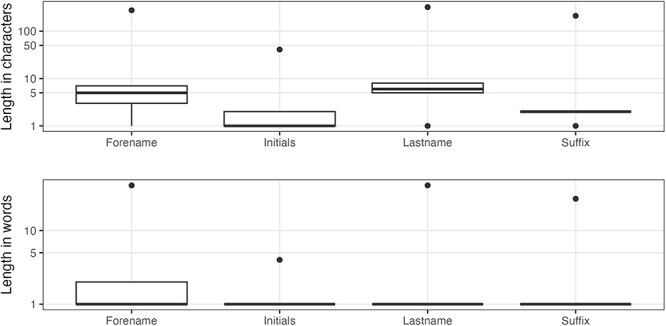
Author name character/word distributions.

The most common last names in MEDLINE are Romanized Chinese names (Table 2), which can be very challenging to disambiguate. Looking at the length characteristics (Figure 4), there are a few obvious problems, namely, pollution of the author elements by incorrectly entered collective names (Table 3) and single character last names potentially caused by name transposition errors (Table 4), which is the case for PMID 31812534 (e.g. Potter L).

**Table 2.** Top 10 LastName values

LastName	Occurrences
Wang	1 086 073
Li	895 976
Zhang	878 544
Chen	722 753
Liu	703 743
Lee	547 636
Kim	523 687
Yang	433 439
Wu	360 532
Huang	309 375

**Table 3.** Ten longest LastName values

LastName	Length
Endocrinology Genetics And Metabolism Group Pediatric Branch Of Chinese Medical Association Neonatal Screening Group Specialist Committee For Prevention And Control Of Birth Defects Chinese Association Of Preventive Medicine Prevention And Control Committee Of Birth Defects Pediatric Branch Of Chinese Medical Association	322
The Group Of Minimally Invasive Spinal Surgery And Enhanced Recovery Professional Committee Of Orthopedic Surgery And Enhanced Recovery Association Of China Rehabilitation Technology Transformation And Promotion	211
Genetic Disease Society Guangdong Precision Medicine Application Association Prenatal Diagnosis Group Maternal And Child Health Care Society Guangdong Medical Association Expert Committee Of Prenatal Diagnosis	209
Arir Associazione Riabilitatori dell’Insufficienza Respiratoria Sip Società Italiana di Pneumologia Aifi Associazione Italiana Fisioterapisti And Sifir Società Italiana di Fisioterapia E Riabilitazione	201
This Paper Is A Co-Publication Between European Journal Of Preventive Cardiology European Heart Journal Acute Cardiovascular Care And European Journal Of Cardiovascular Nursing	176
Committee For Birth Defect Prevention And Control Chinese Association Of Preventive Medicine Genetic Testing And Precision Medicine Branch Chinese Association Of Birth Health	174
Consensus Group Of Experts On Application Of Metagenomic Next Generation Sequencing In The Pathogen Diagnosis In Clinical Moderate And Severe Infections	152
Expert Committee Of The Inter-Laboratory Quality Assessment Of Prenatal Screening And Diagnosis Clinical Test Center Of The National Health Commission	150
For The Antimalarial Therapeutic Efficacy Monitoring Group National Malaria Elimination Programme The Federal Ministry Of Health Abuja Nigeria	142
On Behalf Of The Association Of Rural Surgeons Of India-Lancet Commission On Global Surgery Consensus Committee Arsi-LCoGS Consensus Committee	142

**Table 4.** Top 10 shortest LastName values

LastName	Occurrences
S	756
A	704
E	636
M	592
O	563
K	497
R	453
P	363
G	306
V	279

The author forename field is 99.913% complete. Regarding the length, before 1945, the longest value in the forename field was three characters long, which reflects the policy to only hold author initials. The distributions, in Figure 4, clearly show that there are outliers. As shown in Table 5, these are primarily for working groups (a validity error), but the first row represents a different form of data preparation error where the affiliation has been concatenated with the forename.

**Table 5.** ForeName values over 100 characters

PMID	LastName value	ForeName value	Length
34313229	Choi	Moon Hyung Department Of Radiology Eunpyeong St Mary’s Hospital College Of Medicine The Catholic University Of Korea Seoul Republic Of Korea Catholic Smart Imaging Center Eunpyeong St Mary’s Hospital College Of Medicine The Catholic University Of Korea Seoul Republic Of Korea	276
33145749	En Representación Del Grupo de Trastornos de la Conducta Y Del Movimiento Durante El Sueño de la Sociedad Española de Sueño	En Representación Del Grupo de Trastornos de la Conducta Y Del Movimiento Durante El Sueño de la Sociedad Española de Sueño	123
32329046	En Representación Del Grupo de Estudio de Enfermedades Desmielinizantes de la Comunidad Autónoma de Madrid	En Representación Del Grupo de Estudio de Enfermedades Desmielinizantes de la Comunidad Autónoma de Madrid	106
32433836	Pharmakopsychiatrie	The Therapeutic Drug Monitoring Task Force Of The Arbeitsgemeinschaft Für Neuropsychopharmakologie Und	102

The author initials field has a completeness of 99.912%. The first record in Table 5 also has the distinction of being the longest initials value at 41 characters long (the outlier in the top part of Figure 4). Other longer initials fields may be the result of collective names, be the repetition of the forename or run together author names in the forename field (e.g. PMID 31646832).

Completeness does not apply to author suffixes since not everyone has a suffix to their name. In terms of uniqueness, there are 823 distinct values across 483 541 entries. There are also consistency issues, examples of which can be observed in Table 6 (e.g. Jr, Junior and Júnior). Figure 4 shows the range of suffix lengths and clearly indicates that there is something wrong with at least some records. When we look at the longest values for author suffixes (Table 7) and the most common single character values (Table 8), it becomes clear that there are multiple data issues related to the author suffix field; the general theme of misplaced values, or value ‘pollution’, occurs across fields and is a major data quality weakness for the MEDLINE records.

**Table 6.** Top 15 suffixes

Suffix value	Occurrences
Jr	374 510
3rd	74 260
2nd	20 364
4th	5828
Sr	4075
Junior	535
Júnior	380
Filho	241
PhD	238
5th	204
Neto	200
III	199
Dr	146
6th	129
MD	99

**Table 7.** Ten longest suffixes

Suffix value	Length
Brian Buckley Caitlin Cornell Alyssa Fuller Eric Hojnowski Ryan LaFollette Yelena Livshits Todd Michaelis Claire Motyl Tarakad Ramachandran Devan Rahmachandrin Sofia Seckler Evaline Tso And Kate Zmijewski-Mekeem	211
European Society Of Clinical Microbiology And Infectious Diseases Escmid Vaccine Study Group Evasg	98
(Conceptualization; Review and editing; Read and approved final version of manuscript)	86
Faculty of Bioscience and Bioindustry, Tokushima University, Tokushima, Japan	77
BA, MBBS (Hons), FRANZCP, PhD, Dip Psychodynamic Psychotherapy, Cert ATP	72
on behalf of the Portuguese visual impairment study group (PORVIS-group)	72
(Writing original draft; Read and approved final version of manuscript)	71
RN, Cert Psych Nurs, BA (Hons), Dip Ed, B Ed, M Ed, PhD, FACMHN	63
DVM, PhD, Diplomate ABVP (Dairy Practice), SFHEA, NVS, MRCVS	60
B Phil (Hons), B Soc & Comm Stud (Community Development)	60

**Table 8.** Top 10 shortest suffixes

Suffix value	Occurrences
*	32
S	12
K	11
W	11
J	8
F	8
†	8
A	7
P	7
M	5

The PubMed DTD does not have a dedicated field for an electronic mail (e-mail) address. From 1996, NLM included ‘the first author’s e-mail address at the end of <Affiliation>, if present in the journal. Furthermore, as of 1 October 2013, NLM no longer edits affiliation data to add e-mail address’ (14).

A word of caution about relying on e-mail addresses as a discriminator for author name disambiguation, note that the most common email address is user@example.com, which occurred 2023 times in the MEDLINE dataset of this study. Additionally, there are other non-specific email addresses such as journal editorial mailboxes.

Since 2010, the PubMed DTD has included an Identifier element, which has been used from 2013 (14). However, it has <3% completeness of author records (Table 9) and it is worth noting that there are occurrences where the same ORCID identifier has been incorrectly allocated to multiple authors within a paper.

**Table 9.** Author ORCID measures

Identifier	Completeness (%)	Validity (%)	Uniqueness (%)
ORCID	2.820	99.915	40.921

### Data quality issues related to affiliation names

**Figure 5.**
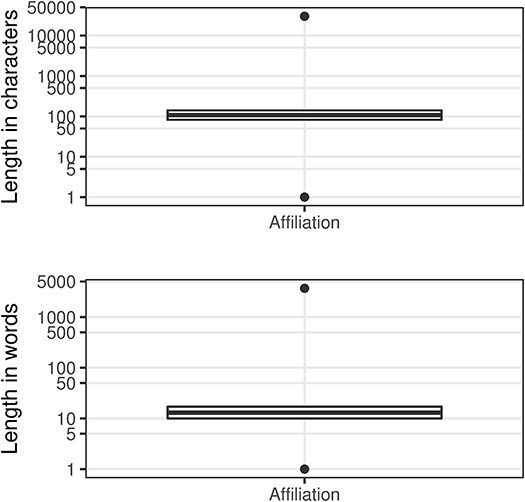
Affiliation character/word distributions.

An author’s institutional affiliation is a very important information field, but the completeness is only around 42%. We have not derived a validity score, but there are quality problems within that set that are obvious from the length distributions (Figure 5). As previously mentioned, this field may contain values that are not written in English as well as non-american standard code for information interchange characters.

**Figure 6.**
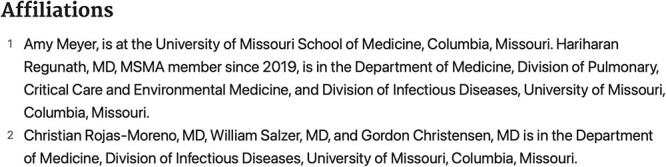
An example of narrative affiliations.

In Figure 5, the outliers at the top of the range, which we have termed ‘narrative affiliations’, typically describe the affiliations for many, if not all, of the contributors to the paper (e.g. see Figure 6 where we show the entry from the article with PMID 32308221). These narrative affiliations may also be repeated for all the author entries within the author list. At the other end of the range, there are many incomplete or indistinguishable entries (Table 10).

**Table 10.** Top 15 affiliations under 20 characters long

Affiliation string	Occurrences
.	5761
,.	2463
London, UK.	601
Editor-in-Chief.	468
London.	405
Pathology.	360
GSK, Siena, Italy.	342
Duke University.	341
Harvard University.	332
McGill University.	329
Paris, France.	323
School of Medicine.	303
Yale University.	301
Editor.	295
Radiology.	262

Our parsing has not included any special case exclusions. We note that ‘pubmed_parser’ (15) excludes ‘For a full list of the authors’ affiliations please see the Acknowledgements section.’—although this exact string only occurs once within our selected dataset of over 51 million affiliation strings! It should also be noted that ‘as of 1 October 2013, NLM no longer performs quality control of the affiliation data’ (14).

While multiple affiliations were possible from the 2015 DTD (14), this is a good place to mention how some data providers concatenate multiple affiliations for an author in a single element. Here is an example for Yong-Beom Park (PMID 29465366):

‘Division of Rheumatology, Department of Internal Medicine, Yonsei University College of Medicine, Seoul; and Institute for Immunology and Immunological Diseases, Yonsei University College of Medicine, Seoul, Republic of Korea’.

Affiliation identifiers, such as ISNI and Global Research Identifier Database (GRID), were possible from the 2015 DTD (14). We have captured values for those too in Table 11. Note that the GRID dataset was transitioned to the Research Organization Registry (ROR) (34), although ROR identifiers were not present in the MEDLINE data at the time of the study.

**Table 11.** Key measures for affiliations/affiliation identifiers

Identifier	Completeness (%)	Validity (%)	Uniqueness (%)
ISNI	0.002	99.965	22.803
GRID	0.003	100.000	23.752
Affiliation	42.526	N/A	45.979

### Data quality issues related to articles

#### Article persistent identifiers

As can be seen in Table 12, the application of DOI, although not perfect, reaches a respectable score in terms of uniqueness, but there are issues with the validity of those identifiers and a significantly low score in terms of completeness; we will examine the impact that earlier publications have on DOI completeness.

**Table 12.** MEDLINE article identifiers

Identifier	Completeness (%)	Validity (%)	Uniqueness (%)
DOI	71.373	99.377	99.949

#### Publication year

The PubDate element contains separated date components ‘for the great majority of records’ (14); however, it also has a fallback MedlineDate for ‘when parsing for the separate fields is not possible’ (2.1 million occurrences). For instance, the MedlineDate element may describe time spans (e.g. ‘1946 May–June’ from PMID 20292550). Of the 1% of MedlineDate instances that describe a time span, the largest by some margin is 28 years (PMID 12125658), based on the difference between the four-digit value extracted by a regular expression using a greedy quantifier (longest substring match) and that extracted using a reluctant quantifier (shortest substring match). Regular expression (with either reluctant or greedy quantifiers) extraction typically results in a viable publication year, but the cases in Table 13 complicate extraction of the publication year. The first two examples include the journal pagination range, and consequently, the MedlinePgn element contains ‘Unknown’, whereas the third example is clearly wrong. This class of problem is not apparent from the front end of PubMed, but affects bulk processing of the XML data.

**Table 13.** Example of erroneous MedlineDate values

PMID	MedlineDate
11662976	1975 part 2): 1125–1132, Dec
11665278	1980 Suppl): 1035–15 August 1041
32422596	1^b^

b Subsequently revised from <MedlineDate>1</MedlineDate> to <Year>2020</Year><Month>11</Month>.

**Figure 7.**
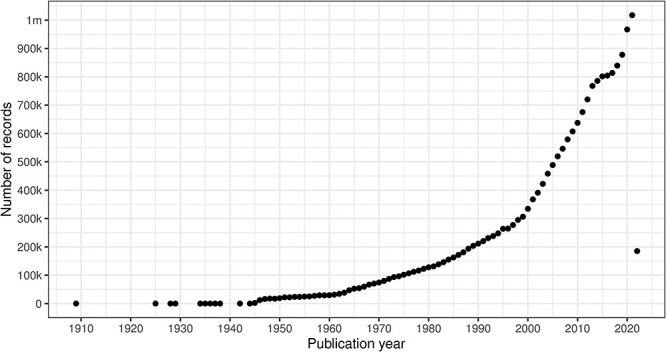
Count of citation records with a valid DOI per publication year (excluding erroneous years).

Figure 7 illustrates the volume of citation records with a valid DOI per publication year with 2022 in progress. Note that as of Q1 2022, there are not yet articles scheduled for publication in subsequent years.

#### Abstract

**Figure 8.**
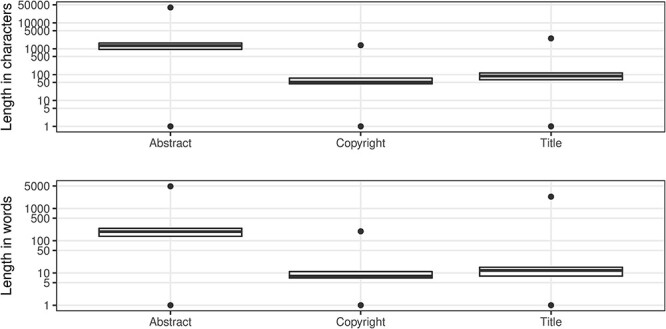
Article character/word distributions.

The abstract field was added to the PubMed record in 1975 (14) and ‘in the absence of a formally labeled abstract in the published article, text from a substantive’ summary, ‘summary and conclusions’ or ‘conclusions and summary’ may be used (14). The abstract text, which may be subject to copyright restrictions, is a prime candidate for text mining. Consequently, for the two-thirds of the records with an abstract, it is useful to understand their length distribution (Figure 8) in order to evaluate their utility for text mining applications. While the uniqueness is 99.942%, there is still a significant number (over 11 thousand abstracts) with non-unique abstract values. From the length information, we can infer that these abstracts provide minimal context for text mining applications toward the lower end of these ranges, as seen in Table 14.

**Table 14.** Top 15 abstracts under 20 characters long

Abstract text	Occurrences
[Figure: see text].	579
.	182
Not available.	106
N/A.	51
n/a.	50
no summary.	48
Null.	41
NA.	29
No Abstract.	22
&lt; p/&gt;.	20
Editorial.	17
EDITORIAL.	16
	13
No abstract.	13
None.	10

**Figure 9.**
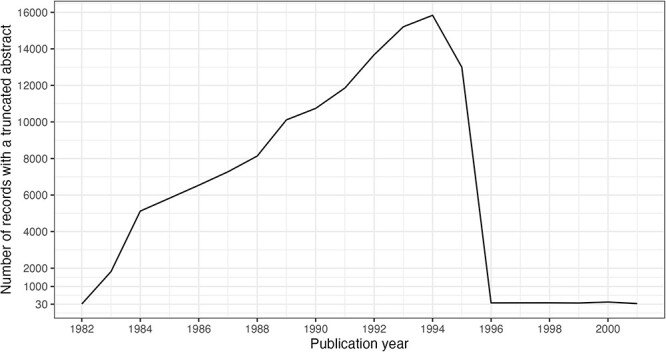
Truncated abstracts by publication year. Filtered to include years with ≥30 occurrences.

It is noteworthy that 125 916 abstracts, <1% of present abstracts, have been subject to truncation due to data entry policies. The truncation limits were 250 words, then 400 words and then 4096 characters in 1996, which was raised to 10 000 characters in 2000 (14). The impact of the 1996 policy change is clearly visible in Figure 9.

#### Copyright

An important consideration when mining MEDLINE should be whether copyrighted material is being used. The NLM terms and conditions clearly state that they do not provide legal advice (35). The copyright information field was introduced in 1999 (14), with a completeness measure of almost 22% of the records that have an abstract. From Table 15, it is evident that Elsevier is most consistent in supplying copyright statements although there is some lack of consistency regarding the actual values. Figure 8 shows the distributions of character length and word tokens; it should be clear that at the low end of the range, there must be some invalid values (Table 16).

**Table 15.** Top 10 copyright statements

Copyright information	Occurrences
Copyright © 2020 Elsevier Inc. All rights reserved.	40 773
© 2021. The Author(s).	39 577
Copyright © 2020 Elsevier B.V. All rights reserved.	39 221
Copyright © 2020 Elsevier Ltd All rights reserved.	39 220
Copyright © 2016 Elsevier Inc. All rights reserved.	38 600
Copyright © 2019 Elsevier Inc. All rights reserved.	37 672
Copyright © 2018 Elsevier Inc. All rights reserved.	37 414
Copyright © 2017 Elsevier Ltd All rights reserved.	36 833
Copyright © 2017 Elsevier Inc. All rights reserved.	36 817
Copyright © 2018 Elsevier Ltd All rights reserved.	36 766

**Table 16.** Top 10 short copyright statements

Copyright information	Occurrences
© 2013.	6941
excerpt	4996
© The author(s).	3193
© FASEB.	1444
full text	1238
©2011 AACR.	1159
©2013 AACR.	1145
©2012 AACR.	958
Celsius.	956
© 2017 The Authors.	925

#### Title

MEDLINE has just over 7500 records without an ArticleTitle element, leading to a completeness value of 99.974%. The uniqueness of the title field is approaching 98%. Like our observations for the abstracts, there are standard article titles that relate to the publication type toward the lower end of the character length and the number of word token ranges (Figure 8; see also Table 17). While these are not strictly errors, typically representing long established journal structure or public discourse conventions, they make it much harder to extract meaning and value from the bibliographic record alone.

**Table 17.** Top 15 article titles under 20 characters long

Article title	Occurrences
[Not Available].	13 440
Reply.	1972
Invited commentary.	1896
Editorial comment.	1676
Editorial.	1465
Response.	1312
Discussion.	1052
Editorial Comment.	1051
Preface.	974
The authors reply.	768
In reply.	714
Introduction.	585
In Reply.	519
Authors’ response.	469
Foreword.	428

#### Language

Another important consideration for text mining is the language, or languages, that the article is published in. It should be noted that PubMed includes translated titles, in square brackets, where appropriate. The language element contains language codes from the US Library of Congress MARC (36) schema, such as ‘chi’ for Chinese. The language code table (37) includes ‘und’ for undetermined and ‘mul’ for multiple languages. However, language codes can also be concatenated together; for example, ‘fregerita’ means that the article was published in French, German and Italian.

The language field is complete for the entirety of the MEDLINE records, but if we treat a solitary value of ‘und’ or ‘mul’ (238 470 and 1399 occurrences, respectively) as invalid, then the validity of this field is 99.55%. This excludes cases where they are present with other values too. From a recency perspective, ‘und’ last occurred in 2002 and that is the only occurrence since 1985; ‘*mul*’ occurred once in both 2016 and 2015, but before that, it was last seen in 2011.

The maximum number of languages specified for a record is 6, but the 75th percentile is 1. Considering the values individually by splitting the strings and exploding the resulting array allows us to produce the top 10 languages (Table 18). Note that almost 84% of the records within the MEDLINE sample are published in English. The next most common language, German, only accounts for ∼3% of articles.

**Table 18.** Top 10 languages

Language code	Occurrences
eng	24 290 379
ger	861 109
fre	744 111
rus	697 806
jpn	429 283
spa	364 920
chi	329 153
ita	305 526
und	239 588
pol	172 956

### Data quality issues related to journals

The key identifier provided in MEDLINE for a journal is the US NLM identity. When compared to the J_MEDLINE reference dataset of MEDLINE-indexed journals (8), the NLM identifiers have a ‘referential’ integrity (18) measurement of 99.989%. There were 146 NLM identifiers that were not included within the J_MEDLINE dataset, affecting 3045 articles. When considering a graph representation of the dataset, this would result in dangling edges that may not be permitted by some graph storage engines, such as Neo4j.

### Data quality issues related to time evolution

**Figure 10.**
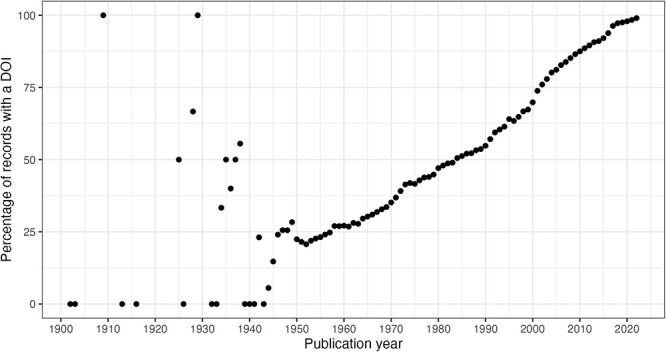
DOI percentage of articles per publication year.

**Figure 11.**
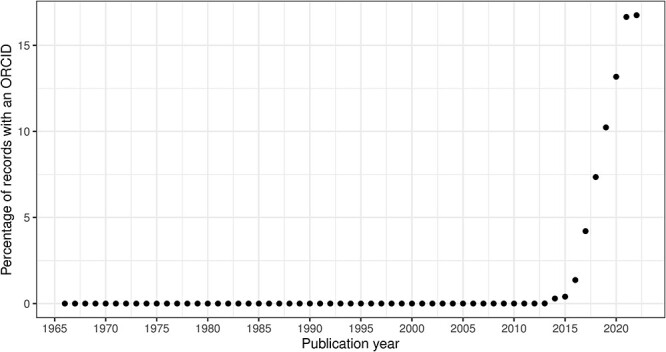
ORCID percentage of authors per publication year.

**Figure 12.**
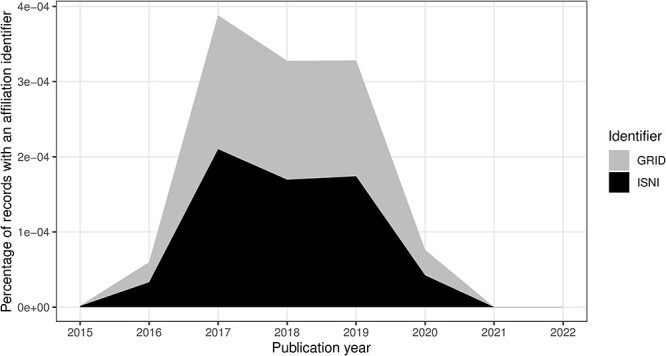
ISNI and GRID percentage of authors per publication year.

**Figure 13.**
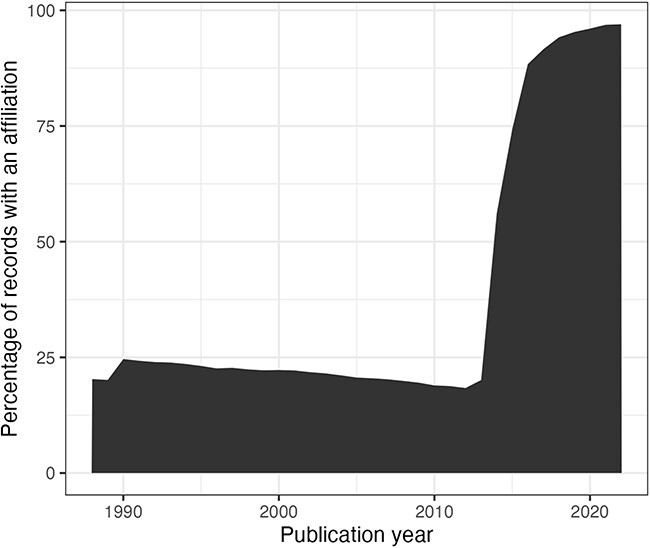
Percentage of authors per publication year (from 1988) with an affiliation string.

In this section, we consider the change over time for some of the key identifiers. Are there any obvious trends in whether identifiers are becoming more pervasive or prevalent in newer citation records? For articles, the answer is clearly yes; DOIs are almost ubiquitous for new articles (Figure 10); for authors, ORCIDs have been on the rise to just under 17% of authors per year (Figure 11); however, affiliations do not follow the same trend as both GRID and ISNI usage peaked in 2017, having first appeared in records from 2015 (Figure 12). This leaves us with the tedious task of disambiguating the affiliation of the authors in the records. As can be seen in Figure 13, the vast majority of recent records contain an affiliation string for all authors; this is due to a policy change in 2014 to collect affiliations for all contributors (14).

## Conclusions

PubMed is an enormously valuable resource for the biomedical sciences and healthcare; yet, those attempting to identify authors and affiliations, or otherwise use the records from that database, need to be aware of the quality issues within the dataset. This article has highlighted some of those data quality concerns.

The data are subject to many human errors, such as typographical errors, and system-related errors, such as inconsistent representations of author names (leading to the synonym problem) and affiliations. There is a lack of author identifiers (contributing to the homonym problem) and a significant lack of affiliation identifiers. Being an aggregated source, the PubMed database suffers from multi-source problems such as inconsistent representations from the upstream XML data providers that result in a high degree of lexicographic entropy.

In summary, our work supports the following conclusions:

- Given the incompleteness and uniqueness of identifying fields, the disambiguation of author names remains a significant problem for PubMed, particularly for records dating before 2014. Publishers need more robust controls in place for ensuring that high-quality author information is present during manuscript submission.
- PubMed has excellent integrity for NLM-internal identifiers (e.g. MeSH), although there is the noted exception around the J_MEDLINE dataset. Beyond the NLM data items, the majority of articles are labeled with a DOI and the DTD provides support for identifiers for authors and institutions, both of which are far from complete. The DTD also caters for grant information and auxiliary data through the DataBank elements, although these were beyond the scope of our work.
- Overall, there is an improvement in the use of identifiers; in particular, records created since 2015 exhibit an increase in external identifiers. However, the data quality for institutional identifiers is poor and their use has been diminishing over time.

Unless the data quality issues are addressed retroactively, they will weaken (if not entirely distort) any subsequent data analysis. While publishers detect some errors and revise the records, perhaps, an intervention in current publishing systems, to prevent the upstream data sources of PubMed from manifesting the data quality issues mentioned herein, is the best one can hope for the future. Much like the application of machine learning has been applied within the NLM for indexing (e.g. with the Medical Text Indexer tooling (38)), the NLM could enhance their process with systems that possess a learning architecture to improve and accelerate the curation of the PubMed records. It is also possible that another information provider will provide an open data repository containing cleansed PubMed data, although a proprietary offering is more likely.

Another possibility for better use of the PubMed treasure trove is the creation of an open-source library for cleansing the data, or at least properly identifying the data quality issues, and optimizing the amount of information that one can obtain from processing the PubMed records. Once this is accomplished with one programming language, the open-source community can augment the library and expand its adoption in other programming languages, for example, by porting the library.

Lastly, the community would benefit from the availability of open-source libraries that can accurately perform author name disambiguation or a substantial set of ‘gold data’ released under a permissive license that can be used for training and validation; that dataset, however, should be orders of magnitude larger than the ones that are currently available (e.g. the ‘amorgani/AND’ dataset (39, 40)). Here, Torvik and Smalheiser’s ‘Author-ity 2018’ dataset (41) representing 29.1 million PubMed articles and 114.2 million author name instances, released under a Creative Commons Attribution license, could provide a robust benchmark for evaluation.

## Data Availability

The PubMed data files are available to download from https://ftp.ncbi.nlm.nih.gov/pubmed/. The derived data generated in this research will be shared on reasonable request to the corresponding author.
